# Successful External Cephalic Version: Factors Predicting Vaginal Birth

**DOI:** 10.1155/2014/860107

**Published:** 2014-01-22

**Authors:** Pei Shan Lim, Beng Kwang Ng, Anizah Ali, Mohamad Nasir Shafiee, Nirmala Chandralega Kampan, Nor Azlin Mohamed Ismail, Mohd Hashim Omar, Zaleha Abdullah Mahdy

**Affiliations:** ^1^Department of Obstetrics and Gynaecology, UKM Medical Centre, Malaysia; ^2^Department of Obstetrics and Gynaecology, UPM, Malaysia

## Abstract

*Purpose*. To determine the maternal and fetal outcomes of successful external cephalic version (ECV) as well as factors predicting vaginal birth. *Methods*. The ECV data over a period of three years at Universiti Kebangsaan Malaysia Medical Centre (UKMMC) between 1 September 2008 and 30 September 2010 was reviewed. Sixty-seven patients who had successful ECV were studied and reviewed for maternal, fetal, and labour outcomes. The control group comprised patients with cephalic singletons of matching parity who delivered following the index cases. *Results*. The mean gestational age at ECV was 263 ± 6.52 days (37.5 weeks ± 6.52 days). Spontaneous labour and transient cardiotocographic (CTG) changes were the commonest early adverse effects following ECV. The reversion rate was 7.46%. The mean gestational age at delivery of the two groups was significantly different (*P* = 0.000) with 277.9 ± 8.91 days and 269.9 ± 9.68 days in the study group and control groups, respectively. The study group needed significantly more inductions of labour. They required more operative deliveries, had more blood loss at delivery, a higher incidence of meconium-stained liquor, and more cord around the neck. Previous flexed breeches had a threefold increase in caesarean section rate compared to previous extended breeches (44.1% versus 15.2%, *P* = 0.010). On the contrary, an amniotic fluid index (AFI) of 13 or more is significantly associated with a higher rate of vaginal birth (86.8% versus 48.3%, *P* = 0.001). *Conclusions*. Patients with successful ECV were at higher risk of carrying the pregnancy beyond 40 weeks and needing induction of labour, with a higher rate of caesarean section and higher rates of obstetrics complications. Extended breech and AFI 13 or more were significantly more likely to deliver vaginally postsuccessful ECV. This additional information may be useful to caution a patient with breech that ECV does not bring them to behave exactly like a normal cephalic, so that they have more realistic expectations. However, these predictive factors needed further confirmation and hopefully, in the future, they would be able to further enhance counselling prior to ECV.

## 1. Introduction

Publication of the International Randomized Term Breech Trial in 2000 [1] appeared to confirm the presumption that primary caesarean section would reduce morbidity and mortality among children in breech presentation at term. It brought about a major change in clinical practice worldwide. The Term Breech Trial, however, had a major impact on the attitude of obstetricians towards vaginal breech delivery [2] as the number of caesarean sections for breech presentation at term increased considerably over the following years and is still on the rise. This increased rate of caesarean section might accelerate the associated maternal morbidity related to anaesthesia or operative interventions. It also placed subsequent pregnancies and labour at a higher risk.

In order to control this rise in caesarean section rate obstetricians seriously looked into external cephalic version (ECV) as a method that can safely reduce the incidence of breech presentation at term [3, 4]. Both the American College of Obstetricians and Gynaecologists (ACOG) and British Royal College of Obstetricians and Gynaecologists (RCOG) recommended the use of ECV as first line management of breech presentations at term [5, 6].

Although the sole intent of ECV was to reduce the overall caesarean sections for breech presentation, there is still overwhelming controversy as to whether this aim is ultimately achieved [7]. To date, there are conflicting evidences, as some studies had shown an increased rate of caesarean delivery even after a successful ECV [8], whereas others did not [9–11]. Some studies also quoted an increase in instrumental and overall obstetrics intervention following a successful ECV [8]. Therefore, this 3-year audit aimed to concentrate our review on the maternal, fetal, and labour outcomes following successful ECV at Universiti Kebangsaan Malaysia Medical Centre (UKMMC). We also aimed to explore the factors predicting successful vaginal birth following ECV.

## 2. Materials and Methods

### 2.1. Study Design

A retrospective review was carried out on ECV performed over a period of three years between 1 September 2008 and 30 September 2010 at the Department of Obstetrics and Gynaecology, UKMMC. Patient details and outcomes of ECV were obtained from the ECV record book. Case notes for those who had successful ECV were reviewed for maternal, fetal, and labour outcomes. Successful ECV was defined as cephalic presentation at the end of ECV. All successful ECV were reviewed after one week as per hospital protocol to exclude spontaneous reversion. In this review, the study group comprised patients with successful ECV who delivered in UKMMC. Patients lost to follow-up after ECV were excluded from the study.

It is our departmental policy to offer ECV routinely, in the absence of contraindications, to women with breech presentation after 36 weeks. Following a successful version, the management of labour and delivery did not differ from other patients. They were allowed vaginal birth unless subsequent maternal or fetal indications arise necessitating a change in the mode of delivery. Therefore, the antenatal and intrapartum care of patients following successful ECV was governed by the same departmental protocol as for other pregnancies. Induction of labour was performed at 40 weeks plus 10 days of gestation in the absence of any other problems.

Data regarding fetal presentation at the onset of labour, mode of delivery, intra-partum and postpartum events, and neonatal outcome were recorded. Adverse outcome or events including those occurring within 24 hours of ECV were recorded. Adverse outcomes subsequent to this period were also considered; however, these would be more difficult to attribute to the ECV. Intra-partum events and details were documented. Neonatal assessment was performed after delivery. Other additional information pertaining to the ECV success as well as rates of reversion was also collected and analysed.

The controls were the next patients who delivered a normal cephalic singleton with similar (matched) parity. Information was obtained from the labour room delivery record book.

Data collection, storage, and analysis were performed using the Statistical Package for Social Sciences (SPSS) for Windows version 13.0 software. Student's *t-*test was used to compare continuous data and chi-square and Fisher's exact test for categorical data. Statistical significance was set at the 5% level (95% confidence interval).

## 3. Result

There were 22, 25, and 20 cases of successful ECV in 2008, 2009, and 2010, respectively. Hence the total number of ECV performed successfully in this 3-year period was 73. Six cases were lost to follow-up: four delivered in other hospitals and two were untraceable. Therefore, only 67 cases were included in the analysis.

### 3.1. The Successful ECV Group

The success rate of ECV was 51.4% (73/142 cases) over the three years. ECV was performed at a mean gestational age of 263 ± 6.52 days (37.5 weeks ± 6.52 days). The mean sonographic estimated fetal weight at the time of ECV was 2.87 ± 0.29 kg (Table 1). There were 34 (50.7%) flexed breeches and 33 (49.3%) extended breeches. The mean amniotic fluid index (AFI) was 13.77 ± 2.45 cm at ECV with range of 12.1 to 16.0 (46.3%). Posterior placentation (50.7%) was the commonest compared to anterior placentation (32.8%) and fundal placentation (16.4%) (Table 1).

There were five deliveries within 24 hours following successful ECV. Three of them had successful vaginal delivery, while one parturient had an emergency caesarean section for poor progress of labour (Table 2). As for fetal complications, four fetuses had CTG changes following ECV. Three were transient changes and only one had persistent pathological CTG changes. The transient CTG changes were, namely, fetal bradycardia and late decelerations. The CTG subsequently reverted spontaneously back to reassuring traces. The persistently pathological CTG was persistent fetal bradycardia warranting emergency caesarean section. The baby was born 2.84 kg with a low cord pH of 7.115 requiring neonatal intensive care unit (NICU) admission for respiratory distress (Table 2). The baby was discharged well after 3 days.

Out of the 67 cases of successful ECV, five (7.46%) fetuses reverted back to either breech presentation or transverse. All of them presented in labour, between 9 and 24 days after ECV, and had emergency caesarean delivery.

### 3.2. The Successful ECV Group versus the Control Group

A total of sixty-seven parity-matched controls were compared with the ECV group. The maternal age ranged between 19 and 45 years in the ECV group and between 20 and 42 years in the control group, giving rise to no significant difference in the mean age (29.91 ± 4.53 versus 28.84 ± 4.79, *P* = 0.184) in both groups (Table 3).

Gestational age at delivery was significantly higher in the ECV group compared to the control group (277.9 ± 8.91 days versus 269.9 ± 9.68 days, *P* = 0.000). The ECV group also had a significantly higher body mass index (BMI) (26.32 ± 4.42 kg/m^2^ versus 23.26 ± 3.68 kg/m^2^, *P* = 0.000) compared to the control group (Table 3).

A large number of subjects in the ECV group required induction of labour as compared to the control group (31.3% versus 4.5%, *P* = 0.000) (Table 4). Most patients in the control group (95.5%) came in spontaneous labour. The presence of meconium-stained liquor was significantly higher (nearly fold) in the ECV group in comparison to the control group (26.9% versus 3.0%, *P* = 0.000). Cord around neck was seen fairly commonly in the ECV group but none was observed in the control group (13.4% versus 0%, *P* = 0.000).

The rate of successful spontaneous vertex delivery was higher in the control group compared to the study group (71.6% versus 59.7%, *χ* ^2^ = 2.119, *P* = 0.203). Similarly, there were more vaginal births (combined spontaneous vertex delivery and instrumental delivery) in the control group compared to the ECV group (80.6% versus 70.1%, *χ* ^2^ = 1.970, *P* = 0.229). However, these differences were not statistically significant. Obstetrics intervention, that is, combined instrumental delivery and caesarean section, was more frequently observed in the ECV group compared to the control group (40.3% versus 28.4%, *χ* ^2^ = 2.119, *P* = 0.203), but again the difference was not statistically significant. Median blood loss at delivery was higher in the ECV group (300 mL versus 250 mL, *Z* = −1.811, *P* = 0.70), but it was not statistically significant. A number of subjects in the study group had postpartum haemorrhage (9.0% versus 3.0%, *χ* ^2^ = 2.127, *P* = 0.274). However, the difference was not statistically significant (Table 4). There were no significant differences in birth weights, Apgar scores, cord pH values, and gender distribution between the two groups. The number of babies admitted to NICU was similar between the two groups (4.5% versus 3.0%, *P* > 0.05) (Table 5).

### 3.3. Factors Influencing Mode of Delivery in the ECV Group

Among patients in the ECV group who went post-dates (beyond 40 weeks), two-thirds had successful vaginal delivery while a third required caesarean section (Table 6). The rate of caesarean section was higher amongst nulliparae (39.4% versus 20.6%) and those with BMI < 25 kg/m^2^ (35.5% versus 25.0%). Women with anterior placentation had the highest caesarean section rate of 40.9% as compared to 36.4% in women with fundal placentation and 20.6% in women with posterior placentation. Previous flexed breeches had almost a threefold increased risk of caesarean section compared to previous extended breeches (44.1% versus 15.2%, *P* = 0.010). On the contrary, AFI of thirteen or more is significantly associated with higher rates of vaginal birth (86.8% versus 48.3%, *P* = 0.001). Both sonographically estimated fetal weight and birth weight did not predict the mode of delivery. This may be due to fetal macrosomia being an exclusion factor in selecting patients for ECV. Male babies had a twofold higher caesarean section rate compared to female babies (40.6% versus 20.0%), but this was not statistically significant. Among all the above-tabulated factors, only extended breech and AFI ≥ 13 at ECV were found to be statistically significant in predicting a successful vaginal birth (Table 6).

## 4. Discussion

Clinical audit is an effective quality improvement process to evaluate important clinical issues [12]. Among the popular topics of concern and controversy in obstetrics practice is the management of breech presentation at term. Breech presentation has become an outstanding issue in the past decade due to its marked contribution to the rising caesarean section (CS) rate, especially as a repercussion of the Term Breech Trial. This retrospective review aimed to analyse the outcome following successful ECV in our hospital. The success rate of ECV over these three years was 51.4%, which has improved compared to those reported in previous local studies [13, 14]. Hofmeyr et al. demonstrated a similar success rate of ECV (40–50%) in routine clinical practice [15]. Conversely, Lau and Ben-Arie et al. reported higher success rates of 69.5% and 78.7% [16, 17].

Amongst the 67 women with successful ECV, the commonest complications occurring within 24 hours of ECV were spontaneous labour (6.0%) and transient CTG changes (4.5%). This is similar to the report by Lau et al. in which transient CTG changes occurred at a rate of 3.3% in their series of 243 cases of ECV [16]. The median interval between ECV and delivery was 15.0 days [8, 18]. Out of the 67 subjects with successful ECV, five fetuses reverted to either breech or transverse (7.46%). This was slightly higher in comparison to other studies where Lau and Feyi-Waboso et al. reported reversion rates of only 4.1% and 3.0%, respectively [16, 19]. The possibility of higher reversion rate could be due to an undiagnosed unstable lie prior to ECV as three of these five cases reverted to an abnormal lie, which required caesarean delivery.

Significantly, more subjects in the ECV group required induction of labour compared to the control group. This was attributable to a larger number of subjects in the ECV group who went beyond 40 weeks (280 days) of gestation, thus increasing the rate of induction of labour. Chan et al. reported a higher rate of induction of labour amongst those with successful ECV but did not show any difference in the rate of prolonged pregnancy [8]. It can easily be postulated that the higher prevalence of prolonged pregnancy with successful ECV is contributed by delayed stretching of the lower uterine segment by the delay in descent of the head into the pelvis. Obviously, the fetal head stretches the lower segment better than the breech. One of the factors determining the length of pregnancy is neurological behaviour of the fetus. Kean et al. suggested that there are neurological differences between term breech fetuses and cephalic fetuses [20]. They demonstrated a significant difference in the number of fetal behaviour state transitions between breech and cephalic fetuses. Although breech fetuses demonstrated similar total length of time in their movements, the sustained movements were shorter. However, the actual impact of this difference remained unclear. They suggested that the failure to sustain activity might be related to underlying neurodevelopmental problem.

Median blood loss at delivery and the prevalence of postpartum haemorrhage were both higher in the ECV group compared to the control group although the differences were statistically insignificant. This may be explained by the more frequent obstetrics interventions and operative delivery in the ECV group.

It was widely recognised that breech presentation was associated with a higher perinatal mortality and morbidity regardless of the mode of delivery. This was supported by Danielian et al. [21], who concluded that breech presentation was a signal for potential fetal handicap. A few studies based in Nigeria documented low 5-minute Apgar scores and unacceptably high perinatal mortality rates in association with vaginal breech deliveries [22, 23]. Our study documented no significant difference between the ECV group and the control group in terms of birth weight, gender, 1- and 5-minute Apgar scores, cord pH values, or rate of admission to the neonatal intensive care unit. However, meconium staining of liquor was found to be nearly ninefold higher amongst the ECV group in comparison to the control group. Again, this may be attributable to a higher prevalence of postdatism in the ECV group (*P* = 0.000). Cord abnormalities were not an uncommon finding amongst the ECV group but none was seen in the control group.

In attempting to identify factors predicting a successful vaginal birth after ECV, it was found that only extended breech and AFI ≥ 13 were statistically significant in this role. This finding is in agreement with that of Zandstra and Mertens who reported that the success rate of ECV in extended breech was higher compared to flexed breech (34.6% versus 14.1%, *P* < 0.05) [24]. In our study, flexed breech contributed slightly higher to successful ECV compared to extended breech (50.7 versus 49.3%); however, post-ECV extended breech favours successful vaginal birth better than flexed breech (44.1% versus 15.2%) (Table 6).

Male fetuses are known to have an increased risk of fetal distress during labour [18] and instrumental as well as caesarean deliveries [25, 26]. This agrees with our finding of male fetuses being more likely to be delivered via CS following successful ECV compared to female (40.6% versus 20%) although the difference was not statistically significant. Possible explanations include differences in intra-partum cardiotocographic changes, higher prevalence of placental dysfunction, and differences in energy metabolism between male and female fetuses [27–29].

The majority of the caesarean deliveries following successful ECV were for fetal distress and labour dystocia (35.0% each). In the control group, 30.7% of caesarean section was for fetal distress and only 15.4% was for labour dystocia. This finding was consistent with results reported by Lau et al. in which there were 2.5- and 2.8-fold increases in intra-partum fetal distress and labour dystocia following ECV [16]. The increased caesarean section and instrumental delivery for fetal distress may be attributed to obstetrician bias, as they are likely to practise a lower threshold for both caesarean section and instrumental delivery for those with successful ECV. Chan et al. [8]; however, did not agree with this explanation. They suggested that fetuses with breech presentation were less tolerant to the stress of labour compared to cephalic fetuses. As for labour dystocia, Vézina et al. [9] proposed a few hypotheses in an attempt to explain the increased incidence of labour dystocia amongst parturients following successful ECV. The proposed hypotheses were as follows: (1) uterine anomalies or atypical maternal pelvis configuration may cause both an increased risk for fetal breech presentation and a higher risk of dystocia during labour, (2) in patients who had a successful ECV, because of the need to have a mobile fetus, it was more likely to have a fetus with an unmoulded, unengaged head or a head in an asynclitic position or a combination of those factors during labour, and (3) patients with successful ECV may have an increased uterine compliance that, in time, can be associated with abnormal uterine contractility resulting in labour dystocia [9].

## 5. Limitations

This was a retrospective review, with 6 patients lost to follow-up. A similar but prospective study with a larger sample size should be conducted in the future to ascertain the findings of this study. As for the estimated blood loss, no standard quantification technique was applied, leading to the possibility of bias.

## 6. Conclusion

Patients with successful ECV were at higher risk of carrying the pregnancy beyond 40 weeks of gestation needing induction of labour. Previous extended breech and AFI 13 or more were significant contributors towards successful vaginal birth following ECV. This additional information may be useful to caution a patient with breech that ECV does not bring them to behave exactly like a normal cephalic, so that they have more realistic expectations. However, these predictive factors needed further confirmation and hopefully, in the future, they would be able to further enhance our counselling prior to ECV.

## Figures and Tables

**Table 1:** Fetal characteristic at external cephalic version (ECV).

	*n* = 67
Sonographic EFW at ECV (kg)	2.87 ± 0.29
Type of breech	
Flexed, sum (%)	34 (50.7)
Extended, sum (%)	33 (49.3)
AFI (cm)	13.77 ± 2.45
AFI (sum, %)	
10.0–12.0	23 (34.3)
12.1–16.0	31 (46.3)
16.1–18.0	9 (13.4)
>18.0	4 (6.0)
Placenta (sum, %)	
Anterior	22 (32.8)
Posterior	34 (50.7)
Fundal	11 (16.5)

All expressed in mean ± SD unless specified.

EFW: estimated fetal weight.

**Table 2:** Complications occurring within 24 hours of external cephalic version (ECV).

Maternal complications		Fetal complications	
Uterine rupture	0	Transient CTG changes	3 (4.5%)
PROM	0	Pathological CTG changes	1 (1.5%)
Abruptio placenta	0	Fetal death within 24 hours of ECV	0
Labour	4 (6.0%)	Fetal death > 24 hours after ECV	0
Emergency LSCS	2 (3.0%)	Others	0

PROM: Prelabour rupture of membrane.

LSCS: Lower segment caesarean section.

**Table 3:** Maternal characteristics of the study group (successful external cephalic version) and control group (spontaneous cephalic presentation).

	Study group (*n* = 67)	Control group (*n* = 67)	*P* value
Age (years)	29.91 ± 4.53	28.84 ± 4.79	0.184
Parity	0.96 ± 1.22	0.94 ± 1.22	
Primigravida (%)	33 (49.3%)	33 (49.3%)	(Matched variable)
Para 1–4 (%)	32 (47.8%)	32 (47.8%)	
Para ≥5 (%)	2 (3.0%)	2 (3.0%)	
Ethnicity			
Malays (%)	46 (68.7%)	50 (74.6)	*χ* ^2^ = 3.033, *P* = 0.387
Chinese (%)	14 (20.9%)	7 (10.4%)	
Indian (%)	2 (3.0%)	3 (4.5%)	
Others (%)	5 (7.4%)	7 (10.5%)	
Gestational age at ECV (days)	263.0 ± 6.52	NA	NA
Gestational age at delivery (days)	277.9 ± 8.91	269.9 ± 9.68	0.000
BMI at delivery (kg/m^2^)	26.32 ± 4.42	23.26 ± 3.68	0.000
Interval of ECV to delivery (median, quartile) (days)	15.0 (8.0, 21.0)	NA	NA

All expressed in mean ± SD unless specified.

**Table 4:** Maternal labour outcome.

	Study group (*n* = 67)	Control group (*n* = 67)	*P* value
Mode of delivery (sum, %)			
Vaginal delivery	40 (59.7)	48 (71.6)	—
Instrumental delivery	7 (10.4)	6 (9.0)	
Caesarean section	20 (29.9)	13 (19.4)	
Type of labour (sum, %)			
Induction	21 (31.3)	3 (4.5)	*χ* ^2^ = 16.445, *P* = 0.000
Spontaneous	46 (68.7)	64 (95.5)	
Estimated blood loss (median, mL)	300.0 (250.0, 300.0)	250.0 (250.0, 300.0)	*Z* = −1.811, *P* = 0.70
Postpartum haemorrhage (sum, %)	6 (9.0)	2 (3.0)	*χ* ^2^ = 2.127, *P* = 0.274
Obstetrics intervention (instrumental delivery and caesarean section) (sum, %)	27 (40.3)	19 (28.4)	*χ* ^2^ = 2.119, *P* = 0.203

**Table 5:** Fetal outcome.

	Study group (*n* = 67)	Control group (*n* = 67)	*P* value
Birth weight (mean ± SD, kg)	3.183 ± 0.377	3.075 ± 0.45	*P* = 0.137
Apgar score (median)			
1 minute	8	9	*Z* = − 1.560, *P* = 0.119
5 minutes	9	9	*Z* = − 1.482, *P* = 0.138
Apgar score 5 minutes <7	0	0	
Cord pH			
Mean ± SD	7.296 ± 0.079	7.309 ± 0.079	*P* = 0.300
<7.2 (sum, %)	8 (11.9)	6 (9.0)	*χ* ^2^ = 0.319, *P* = 0.572
Gender (sum, %)			
Male	32 (47.8)	31 (46.3)	*χ* ^2^ = 0.030, *P* = 0.863
Female	35 (52.2)	36 (53.7)	
Presence of meconium (sum, %)	18 (26.9)	2 (3.0)	*χ* ^2^ = 15.046, *P* = 0.000
Cord abnormality (sum, %)	9 (13.4)	0	*χ* ^2^ = 9.648, *P* = 0.003
NICU admission (sum, %)	3 (4.5)	2 (3.0)	*χ* ^2^ = 0.208, *P* = 1.000

NICU: neonatal intensive care unit.

**Table 6:** Factors predicting successful vaginal delivery following successful external cephalic version.

	Vaginal + instrumental delivery (*n* = 47)	Caesarean section (*n* = 20)	*P* value
Age (years)	29.57 ± 4.26	30.70 ± 5.14	0.356
Gestation at ECV (days)	263.13 ± 6.12	262.85 ± 7.54	0.875
Gestation at delivery			
≤40 weeks/≤280 days	30 (73.1%)	11 (26.9%)	*χ* ^2^ = 0.461, *P* = 0.587
>40 weeks/>280 days	17 (65.4%)	9 (34.6%)	
Interval from ECV to delivery (days)	14.34 ± 8.54	16.20 ± 9.71	0.436
Parity			
Nulliparous	20 (60.6%)	13 (39.4%)	*χ* ^2^ = 2.828, *P* = 0.093
Multiparous	27 (79.4%)	7 (20.6%)	
BMI			
<25 kg/m^2^	20 (64.5%)	11 (35.5%)	*χ* ^2^ = 0.874, *P* = 0.350
≥25 kg/m^2^	27 (75.0%)	9 (25.0%)	
Placenta			
Anterior	13 (59.1%)	9 (40.9%)	*P* = 0.509
Posterior	27 (79.4%)	7 (20.6%)	
Fundal	7 (63.6%)	4 (36.4%)	
Type of breech			
Flexed	19 (55.9%)	15 (44.1%)	*P* = 0.010
Extended	28 (84.8%)	5 (15.2%)	
AFI (cm)	14.15 ± 2.39	12.89 ± 2.44	*P* = 0.054
AFI (cm)			
<13	14 (48.3)	15 (51.7)	*χ* ^2^ = 11.683, *P* = 0.001
13 and >13	33 (86.8)	5 (13.2)	
Sonographic EFW			
<3.0 kg	34 (70.8%)	14 (29.2%)	*χ* ^2^ = 0.038, *P* = 0.846
≥3.0 kg	13 (68.4%)	6 (31.6%)	
Birth weight			
<3.0 kg	16 (80.0%)	4 (20.0%)	*χ* ^2^ = 1.321, *P* = 0.250
≥3.0 kg	31 (66.0%)	16 (34.0%)	
Gender			
Male	19 (59.4%)	13 (40.6%)	*χ* ^2^ = 3.396, *P* = 0.065
Female	28 (80.0%)	7 (20.0%)	

All quantitative data expressed in mean ± SD. EFW: Estimated Fetal Weight.
